# Untargeted metabolomics analysis reveals dynamic changes in co-fermentation with human milk-derived probiotics and *Poria cocos*

**DOI:** 10.3389/fmicb.2022.1032870

**Published:** 2022-12-12

**Authors:** Qishan Wang, Kai Yang, Xinyue Wei, Weicang Qiao, Lijun Chen

**Affiliations:** ^1^School of Bioengineering, Dalian Polytechnic University, Dalian, China; ^2^National Engineering Research Center of Dairy Health for Maternal and Child, Beijing Sanyuan Foods Co. Ltd., Beijing, China; ^3^Beijing Engineering Research Center of Dairy, Beijing Sanyuan Foods Co. Ltd., Beijing, China

**Keywords:** metabolomics, human milk, probiotics, prebiotics, *Poria cocos*, homology of medicine and food

## Abstract

**Introduction:**

To develop functional foods with traditional medicines and homologous food ingredients as well as human milk-derived probiotics, the co-fermentation process of two probiotics, *Lactobacillus plantarum* R9 and *Lactobacillus gasseri* B1-27, isolated from the human milk of healthy parturients and the traditional medicine and food homologous ingredient *Poria cocos,* were separately investigated.

**Results:**

The *Poria cocos* fermentation broth at 2.5% significantly enhanced the total number of *L. plantarum* R9 (*p* = 0.001) and *L. gasseri* B1-27 (*p* = 0.013) after 20 h of fermentation, and Non-targeted metabolomics assays conducted before and after fermentation of the human milk-derived *L. plantarum* R9 and *L. gasseri* B1-27 using the 2.5% *Poria cocos* fermentation broth revealed 35 and 45 differential metabolites, respectively. A variety of active substances with physiological functions, such as L-proline, L-serine, beta-alanine, taurine, retinol, luteolin, and serotonin, were found to be significantly increased. Mannitol, a natural sweetener with a low glycemic index, was also identified. The most significantly altered metabolic pathways were pyrimidine metabolism, pentose phosphate, yeast meiosis, ABC transporter, insulin signaling, and mineral absorption, suggesting that co-fermentation of human milk-derived probiotics and *Poria cocos* may affect the metabolism of trace minerals, sugars, organic acids, and amino acids.

**Discussion:**

Overall, we determined that the optimal concentration of *Poria cocos* to be used in co-fermentation was 2.5% and identified more than 35 differentially expressed metabolites in each probiotic bacteria after co-fermentation. Moreover, several beneficial metabolites were significantly elevated as a result of the co-fermentation process indicating the valuable role of *Poria cocos* as a functional food.

## Introduction

Consumers’ increasing attention toward a healthy diet often leads them to consume functional foods, such as probiotic functional milk beverages rich in plant ingredients (Binda et al., 2020), which can improve metabolic disorders, promote the absorption of trace elements, and strengthen the immune system (Prosekov et al., 2018). Moreover, these functional foods can be used as part of supportive care and be consumed daily to achieve the necessary nutritional balance (Sukhikh et al., 2019). Plant extracts can be used as additives for various purposes in the fermented food industry. They positively affect human metabolic processes in combination with probiotics, improving digestion, cardiovascular activity, and emotional state. Such additives also reportedly exert anti-inflammatory, anti-allergic, anti-bacterial, anti-oxidant, anti-obesity, and anti-diabetic effects (Pothuraju et al., 2015). However, more prospective studies are needed to support the reduction of drug intake by consuming a balanced diet supplemented with functional foods. Food and functional plant materials that are drug homologous are raw materials of nutritional and healthy products and are one of the main categories of dietary supplements.

*Poria cocos* is the dried sclerotia of *Poria cocos* (Schw.) Wolf, a fungus belonging to the family of the polyporaceae, is a well-known traditional herbal medicine in China and other Asian countries since ancient times (Wu et al., 2020). It was recorded in *Shen Nong’s Herbal Classic* that *Poria cocos* is mainly indicated for separating the adverse *qi* from the chest; it can induce urination, tranquilize and replenish the mind after long-time administration, relieve hunger, and prolong the life. Zhongjing Zhang, a famous doctor in the Han Dynasty, created more than 40 prescriptions based on *Poria cocos*, such as “Poria Sini Decoction” and “Wuling Powder.” In the Compendium of Materia Medica, Shizhen Li of the Ming Dynasty also comprehensively discussed the medicinal value and important prescription of *Poria cocos*, which has been administered for more than 2000 years. In addition to medicinal purposes, *Poria cocos* is also a very good nourishing food, which is of great benefit to physical fitness. The earliest record of *Poria cocos* being consumed was an ingredient of the food called “Jade Fragrant Cake” in the “Wu’s Family Feedback” during the Tang Dynasty. *Poria cocos* appears in one of the earliest “lists of varieties that are both food and medicine” published in China (Niu et al., 2012). *Poria cocos* powder, made from the traditional Chinese medicine *Poria cocos*, can be directly swallowed, soaked, or boiled in soup and porridge. It contains triterpenes, polysaccharides, choline, fat, lecithin, potassium, magnesium, and other trace minerals, has many bioactive functions, such as enhancing immunity, lowering blood glucose, resisting inflammation, and inhibiting bacterial growth, improving sleep and reducing swelling, and it is well-known by consumers (Xu et al., 2022). Human milk is the safest food and the main source of essential nutrients for infants. Studies have confirmed the presence of probiotics in human milk, indicating that the intestinal microbiota of infants and young children may be originating from mother’s milk, which indirectly affects their health (Heikkilä and Saris, 2003; Martín et al., 2012). The development of food substances containing human milk-derived probiotics may therefore have a positively impact on the composition of the intestinal microbiota in infants and young children (Prado et al., 2008), and thus of great significance to promote their health (Zhao et al., 2019).

However, changes in people’s traditional eating habits towards consuming higher amounts of commercial snacks with a low nutritional value has contributed to declines in the production and consumption of traditional fermented foods mainly in Asia and Africa, and the diversity of microorganisms used by fermented food suppliers is also declining. It is therefore urgent to explore new fermented food products (Tamang et al., 2016). In the present study, the synergistic interaction between human milk-derived probiotics and the traditional medicinal and food homologous ingredients of *Poria cocos* powder was evaluated. We studied the effects of *Poria cocos* powder on human milk-derived probiotics, and used non-targeted metabolomics to identify dynamic changes before and after the fermentation of human milk-derived probiotics in *Poria cocos* fermentation broth (Mozzi et al., 2013).

## Materials and methods

### Strain activation

The MRS broth was prepared, sterilized at 121°C for 20 min, and then dispensed into 10 ml sterilized test tubes in a BioClean room. The preserved *Lactobacillus plantarum* R9 (Genome Accession Number KU214638.1) and *Lactobacillus gasseri* B1-27 (Genome Accession Number NR113820.1) strains were inoculated into the medium at a volume of 200 μl and placed in an incubator set to 37 ± 1°C for 24 h for anaerobic culture. After confirming the absence of infectious microorganisms using an optical microscope, the broth was activated three times using 2% inoculum to keep the bacteria at high viability for future use.

### Preparation of the *Poria cocos* powder fermentation broth

First, 250 ml of MRS broth liquid medium was prepared. After adjusting its pH to 6.25, the broth was then evenly dispensed into five conical flasks and supplemented with 0, 0.5, 1.25, 2.5, or 3.75 g of *Poria cocos* powder (Beijing Jinchongguang Pharmaceutical Co., Ltd., Beijing, China). After stirring a blank control fermentation broth and four *Poria cocos* fermentation broths at 1, 2.5, 5 and 7.5% were obtained. Subsequently, 10 ml of culture medium in each conical flask was dispensed into five test tubes. After sterilization at 121°C for 20 min, 200 μl of culture medium was separately inoculated with *L. plantarum* R9 and *L. gasseri* B1-27 for culture.

### Total bacterial count

A spectrophotometer (NanoDrop 2000, Thermo Scientific, MA, United States) and a pH meter (Five Easy Plus, Mettler Toledo Instruments (Shanghai) Co., Ltd., Shanghai, China) were used to measure the absorbance (optical density, OD) at 600 nm and the pH value of the fermentation broths with different concentrations of *Poria cocos* at 0, 20, and 24 h after inoculation. Each measurement was repeated three times.

The change in the OD value of each *Poria cocos* fermentation broth was calculated throughout 0–20 h after the onset of the fermentation, using the formula E = ΔOD_experimental group_ − ΔOD_control group_. When E > 0, the *Poria cocos* fermentation broth was considered to have a proliferation-enhancing effect on the probiotics derived from human milk, whereas when E ≤ 0, there was no proliferation-enhancing effect (Lu et al., 2014).

### Preparation of samples for metabolomic analysis

*Poria cocos* fermentation broths with the strongest proliferation enhancement effect were selected from the broths at different concentrations and inoculated with 200 μl of *L. plantarum* R9 and *L. gasseri* B1-27 for 24 h. Appropriate amounts of fermentation broth samples were collected at different stages, transferred into 2 ml centrifuge tubes, supplemented with 500 μl of methanol solution after concentration and desiccation, and vortexed for 1 min. Subsequently, samples were centrifuged at 16992 ×*g* for 10 min at 4°C and the supernatants were collected and transferred into new 2 ml centrifuge tubes for concentration and drying. Samples were reconstituted using 150 μl of 2-chloro-L-phenylalanine solution prepared in 80% methanol–water (Thermo Fishe Scientific, Waltham, MA, United States), filtered through a 0.22 μm membrane, and transferred into a detection bottle for liquid chromatography-mass spectrometry (LC–MS) analysis (Dunn et al., 2011).

### LC analysis

LC analysis was carried out in a Vanquish ultra-high performance LC System (Thermo Fisher Scientific) equipped withthe ACQUITY UPLC^®^ HSS T3 (150 × 2.1 mm, 1.8 μm) column (Waters, Milford, MA, United States). The temperature of the column was maintained at 40°C. The flow rate and injection volume were set to 0.25 ml/min and 2 μl, respectively. For LC-electrospray ionization (ESI)(+)-MS analysis, the mobile phases consisted of (C) 0.1% formic acid in acetonitrile (v/v) and (D) 0.1% formic acid in water (v/v). Separation was conducted using the following gradient: 0–1 min, 2% C; 1–9 min, 2–50% C; 9–12 min, 50–98% C; 12–13.5 min, 98% C; 13.5–14 min, 98–2% C; 14–20 min, 2% C. For LC-ESI (−)-MS analysis, the analytes were separated with (A) acetonitrile and (B) ammonium formate (5 mM) using the following gradient: 0–1 min, 2% A; 1–9 min, 2–50% A; 9–12 min, 50–98% A; 12–13.5 min, 98% A; 13.5–14 min, 98–2% A; 14–17 min, 2% A (Zelena et al., 2009).

### MS conditions

MS detection of metabolites was conducted on a Q Exactive Focus instrument (Thermo Fisher Scientific, MA, United States) using an ESI ion source. Simultaneous MS1 and tandem MS/MS (full MS-ddMS2 mode, data-dependent MS/MS) acquisition was used. The parameters were as follows: sheath gas pressure, 30 arb; aux gas flow, 10 arb; spray voltage, 3.50 kV and − 2.50 kV for ESI(+) and ESI(−), respectively; capillary temperature, 325°C; MS1 range, m/z 81–1,000; MS1 resolving power, 70,000 FWHM; the number of data-dependent scans per cycle, 3; MS/MS resolving power, 17,500 FWHM; normalized collision energy, 30%; dynamic exclusion time, automatic (Want et al., 2013).

### Bioinformatics and statistical analyses

The raw off-machine MS files were converted to mzXML format using the MSConvert tool in the *Proteowizard* software package (v3.0.8789; Smith et al., 2006). The R *XCMS* software package (Navarro-Reig et al., 2015) was used to perform peak detection, peak filtering, and peak alignment processing to obtain a quantitative list of metabolites. The public Human Metabolome Database (HMDB^1^) MassBank^2^, LIPIDMAPS^3^, mzCloud^4^, and Kyoto Encyclopedia of Genes and Genomes (KEGG^5^) databases were used for identifying metabolites. The robust LOESS signal correction (QC-RLSC) was applied for data normalization to correct for any systematic bias. After normalization, only the ion peaks with relative standard deviations (RSDs) of less than 30% were retained to ensure proper metabolite identification.

Principal component analysis (PCA) and orthogonal partial least squares discriminant analysis (OPLS-DA) in the R software package *Ropls* were used to reduce the dimensionality of sample data (Thévenot et al., 2015). The metabolic profiles were visualized as a score plot, where each point represents a sample. The corresponding loading plot and S-plot were generated to provide information on the metabolites influencing sample clustering. All models were evaluated for overfitting using permutation tests. OPLS-DA, which allows discriminating metabolites based on variable importance on projection (VIP) was used. The *p*-value, VIP returned by OPLS-DA, and fold change (FC) were applied to determine the variables that contributed to metabolite classification. Finally, *p* < 0.05 and VIP values >1 were considered statistically significant. The MetaboAnalyst software package (Xia and Wishart, 2011) was used to perform functional pathway enrichment and topology analyses on the identified differentially metabolized molecules. The enriched pathways were used to browse differential metabolites and pathway maps with the KEGG Mapper visualization tool. Agglomerative hierarchical clustering analysis was performed on the dataset using Pheatmap package in R.

## Results

### Effects of different concentrations of *Poria cocos* fermentation broth on the proliferation of human milk-derived probiotics

**Figure 1:**
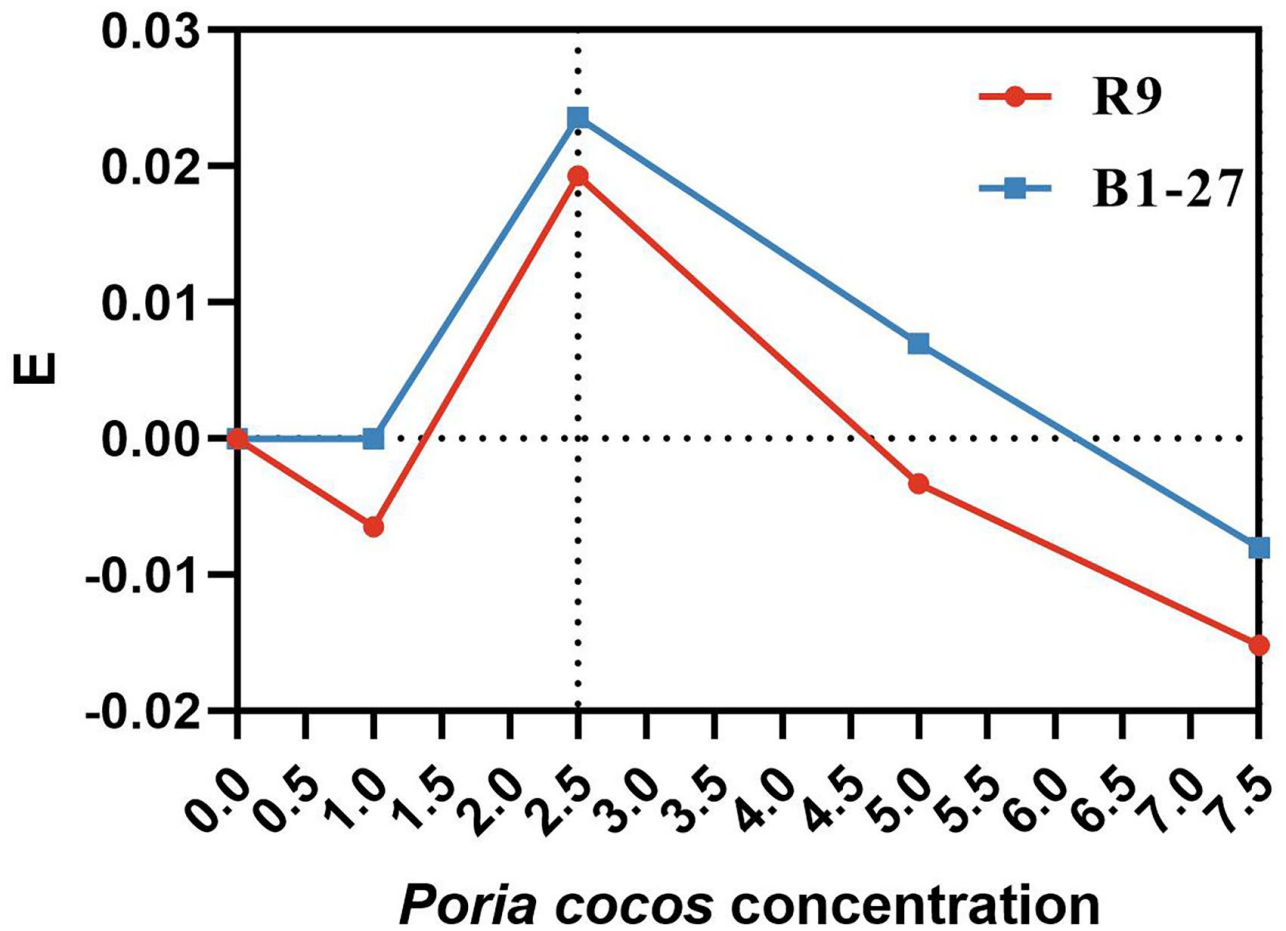
E-values of *Lactobacillus plantarum* R9 and *Lactobacillus gasseri* B1-27 in *Poria cocos* fermentation broth at different concentrations.

The 2.5% *Poria cocos* fermentation broth had the strongest proliferation- enhancing effect on *L. plantarum* R9 and *L. gasseri* B1-27 (Figure 1). The absorbance of the fermentation broth at 600 nm, measured by spectrophotometry, revealed that the total bacterial counts of *L. plantarum* R9 (*p* = 0.001) and *L. gasseri* B1-27 in the 2.5% *Poria cocos* fermentation broth were significantly increased after 20 h of fermentation (*p* = 0.013). In addition, 7.5% *Poria cocos* powder inhibited the growth of both probiotics (Table 1). Moreover, as shown in Table 2, the pH of *Poria cocos* fermentation broths with different concentrations varied between 3.9 and 4.2 during the fermentation of human milk-derived probiotics (Gueimonde et al., 2004), which was in line with the pH value required for commercial fermented milk; however, there was no significant difference between fermentation broths.

**Table 1:** Absorbance (optical density, OD) of the *Lactobacillus plantarum* R9 and *Lactobacillus gasseri* B1-27 fermentation broths with different concentrations of *Poria cocos* fermentation broth.

Concentration of *Poria cocos* fermentation broth	*Lactobacillus plantarum* R9				*Lactobacillus gasseri* B1-27			
	0 h	20 h	24 h	ΔOD\E	0 h	20 h	24 h	ΔOD\E
Control	0.24 ± 0.06	4.37 ± 0.21	4.78 ± 0.03	4.13\0	0.23 ± 0.21	4 ± 0.26	4 ± 0.38	3.77\0
1%	0.49 ± 0.1	3.97 ± 0.2	4.18 ± 0.78	3.48\−0.65	−0.57 ± 0.15	3.2 ± 0.7	3.5 ± 0.53	3.77\0
2.5%	0.77 ± 0.31	6.83 ± 0.38*	5 ± 0.30	6.06\1.93	−0.23 ± 0.55	5.9 ± 0.72*	4.87 ± 0.87	6.13\2.36
5%	0.43 ± 0.59	4.23 ± 0.74	4.23 ± 1.93	3.8\−0.33	0.13 ± 0.15	4.6 ± 1.22	4.77 ± 0.45	4.47\0.7
7.5%	0.21 ± 0.95	2.82 ± 0.81	4.13 ± 0.46	2.61\−1.52	0.83 ± 0.49	3.8 ± 0.31	5.2 ± 0.66	2.97\−0.8

All data in table have been ×100 for conciseness; *indicates *p* ≤ 0.01.

**Table 2:** pH of the *Lactobacillus plantarum* R9 and *Lactobacillus gasseri* B1-27 fermentation broths with different concentrations of *Poria cocos* fermentation broth.

Concentration of *Poria cocos* fermentation broth	*Lactobacillus plantarum* R9			*Lactobacillus gasseri* B1-27		
	0 h	20 h	24 h	0 h	20 h	24 h
Control	6.15 ± 0.02	3.85 ± 0.02	3.64 ± 0.04	6.34 ± 0.03	3.91 ± 0.01	3.89 ± 0.03
1%	6.11 ± 0.03	3.92 ± 0.02	3.87 ± 0.05	6.29 ± 0.02	3.9 ± 0.01	3.86 ± 0.01
2.5%	6.12 ± 0.02	3.98 ± 0.04	3.9 ± 0.03	6.23 ± 0.03	3.89 ± 0.01	3.91 ± 0.04
5%	6.04 ± 0.03	3.96 ± 0.03	3.89 ± 0.01	6.21 ± 0.01	3.91 ± 0.01	3.9 ± 0.03
7.5%	6.03 ± 0.01	3.94 ± 0.02	3.84 ± 0.01	6.17 ± 0.03	3.9 ± 0.02	3.93 ± 0.03

### Metabolites changes after fermentation

**Figure 2:**
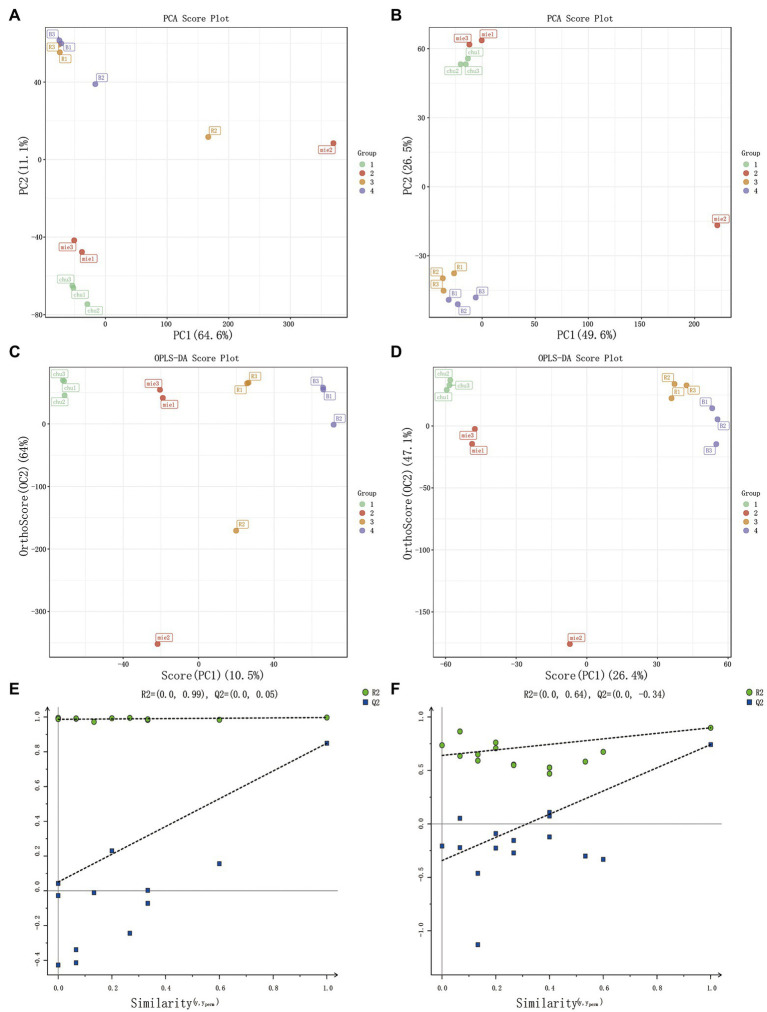
Multivariate statistical analysis of *Poria cocos* fermentation broth. PCA score plot **(A)** in positive EIS mode and **(B)** in negative EIS mode. OPLS-DA score plot **(C)** in positive EIS mode and **(D)** negative EIS mode. OPLS-DA displacement test chart **(E)** in positive EIS mode and **(F)** in negative EIS mode.

The dynamic changes in metabolites at the initial state, after sterilization, and after human milk-derived probiotics co-fermentation with 2.5% *Poria cocos* powder broth were determined using non-targeted metabolomics and analyzed with PCA, OPLS-DA, and other analytical methods. An obvious separation could be observed on the PCA and OPLS-DA score maps (Boulesteix and Strimmer, 2007). Different *Poria cocos* fermentation broths produced clear differences in the positive and negative ESI modes, indicating that the *Poria cocos* fermentation broth of the two probiotic bacteria yielded different metabolites before and after fermentation with a significant classification effect. The total contributions in the positive and negative ESI modes reached 75.7 and 76.1%, respectively, in the OPLS-DA score figure reached 74.5 and 73.5%, respectively, in the OPLS-DA reflecting the metabolite differences between whole samples (Trygg and Wold, 2002). In addition, in the PCA, the R2X in the positive and negative ESI modes were 0.757 and 0.761, respectively; as both values were higher than 0.5, the model had good interpretability. The model built in the OPLS-DA had an R2X of 0.849, RY2 of 0.997, and model prediction ability Q2 of 0.849 in the positive ESI mode and an R2X of 0.735, RY2 of 0.899, and Q2 of 0.742 in the negative ESI mode. The replacement test results showed that the model was stable and reliable, and had good interpretability, validating the good representativeness of the *Poria cocos* fermentation broth samples and the non-targeted metabolomics methodology (Dunn et al., 2011; Xu et al., 2015; Zha et al., 2021; Figure 2).

### Analysis of differential metabolites

**Figure 3:**
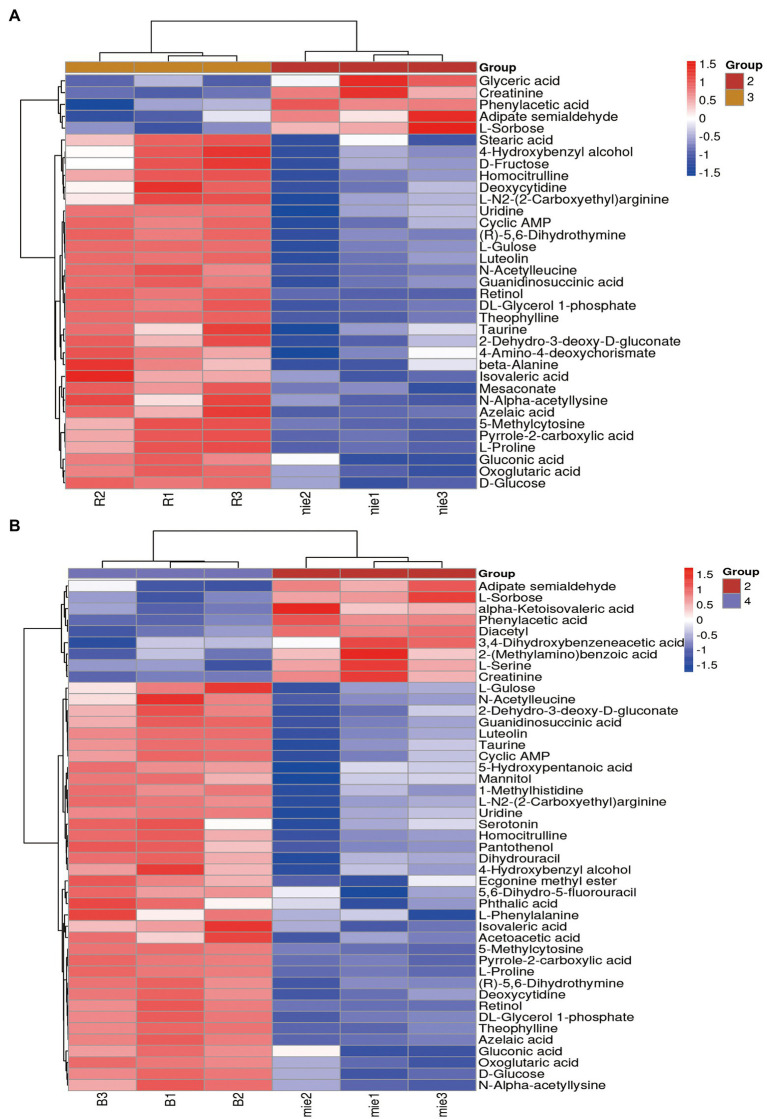
Hierarchical clustering heatmap of bacterial metabolites before and after fermentation. **(A)** Differential metabolites before and after fermentation of *Lactobacillus plantarum* R9. **(B)** Differential metabolites before and after fermentation of *Lactobacillus gasseri* B1-27. Relative contents are indicated as differences in color. The redder the color, the higher the content of the metabolite, and the bluer the color, the lower the content of the metabolite.

Based on the results of the OPLS-DA model, differential metabolites were identified using the cut-off values of *p* < 0.05 and VIP >1 (Dunn et al., 2011), Metabolites were identified based on the precise m/z and retention times of corresponding standard compounds in combination with data queries on public databases such as HMDB. After the initial *Poria cocos* fermentation broth was sterilized by high temperature and pressure, certain organic acid-based metabolomic differences were produced, including 19 differential metabolites such as heptanoic acid, cis-aconitic acid, phthalic acid, and 2-(methylamino) benzoic acid. A total of 35 and 45 differential metabolites were detected in *L. plantarum* R9 and *L. gasseri* B1-27, including organic acids and their substituted derivatives, amino acids and their derivatives, flavonoids, vitamins, purines, and pyrimidines and their derivatives were identified. The differential abundance of these metabolites is displayed as a heatmap in Figure 3. Overall, the contents of various metabolites in the fermentation broth of *Poria cocos* were significantly different before and after fermentation. Generally, the content of metabolites in the *Poria cocos* fermentation broth after *L. gasseri* B1-27 and *L. plantarum* R9 fermentation was lower than that before fermentation, with similar metabolic patterns within each group. The content of organic acids and their substituted derivatives, as well as amino acids and their derivatives, were higher after fermentation than that before fermentation (Figure 3). Comparison of the contents of different metabolites allowed identifying the effects of *Poria cocos* in human milk-derived probiotic fermentation.

### Metabolic pathway analysis of differential metabolites

**Figure 4:**
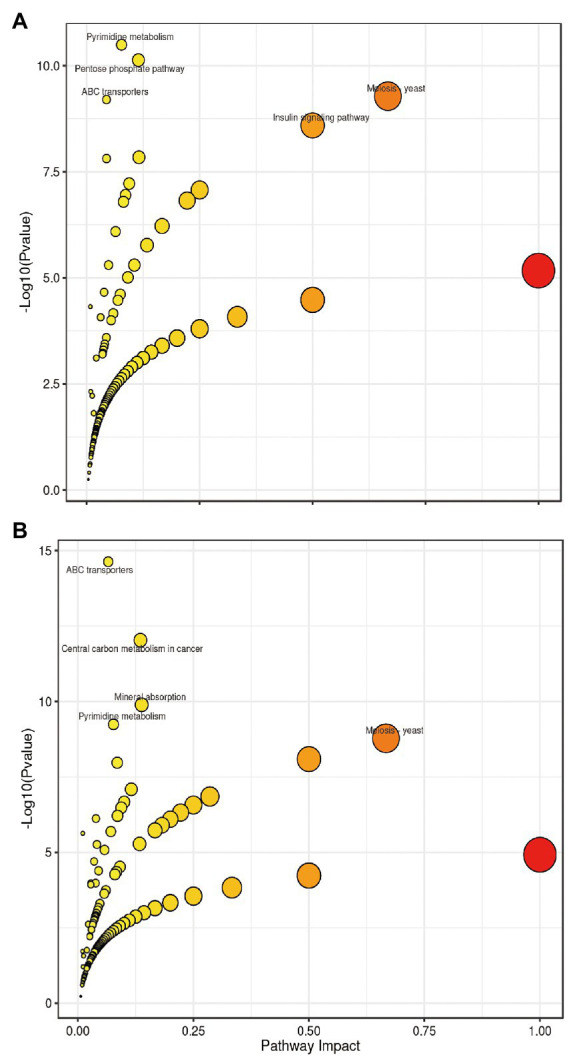
Bubble chart of the metabolic pathways in which the differential metabolites identified after fermentation participate. **(A)** *Lactobacillus plantarum* R9. **(B)** *Lactobacillus gasseri* B1-27. Each bubble represents a metabolic pathway. The impact value of each enriched pathway is displayed in the X-axis and the −log_10_(P) value in the Y-axis. The smaller the *p* value, the more significant the impact of the detected differential metabolites on the pathway is. The larger the impact value, the larger the dot, which means the higher the contribution of the metabolites detected on that pathway, and the greater the impact on the metabolic pathway. Colors are related to *p* values; the lighter the color, the larger the p value.

**Figure 5:**
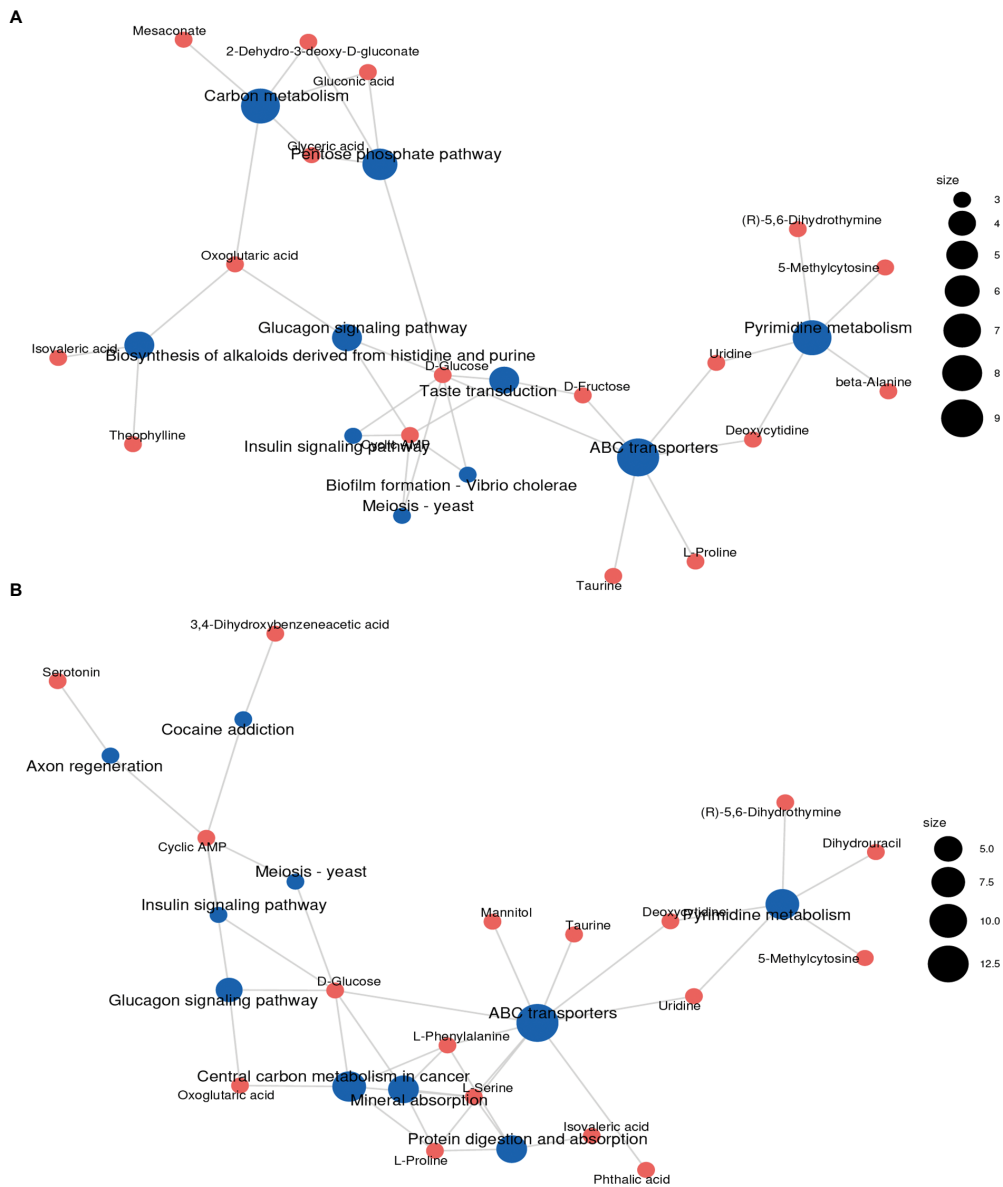
Metabolic pathway association networks. **(A)** After *Lactobacillus plantarum* R9 fermentation. **(B)** After *Lactobacillus gasseri* B1-27 fermentation.

The differential metabolites identified from the co-fermentation broth of *Poria cocos* and *L. plantarum* R9 and *L. gasseri* B1-27 were involved in 165 and 181 metabolic pathways, respectively. The 20 most significant metabolic pathways were determined according to the comprehensive analysis of *p*-values and impact values of each pathway. KEGG enrichment analysis was used to analyze the effect of human milk-derived probiotics on metabolic pathways in *Poria cocos* fermentation broth before and after fermentation, and the results are presented in Figure 4. Five important metabolic pathways were identified, which may also be the key pathways though which human milk-derived probiotics and *Poria cocos* exert their synergistic effects *in vivo*. According to the relative response value of the identified metabolites in the metabolic pathways and the dimension reduction algorithm, the correlation coefficients between the metabolic pathways were calculated and used to draw the metabolic pathway association network presented in Figure 5.

## Discussion

Probiotics are commonly used biological ingredients in many functional fermented foods, and *Lactobacillus* and *Bifidobacterium* species are the most commonly used probiotics. The probiotics used in the present study were *L. plantarum* R9 and *L. gasseri* B1-27, which were isolated from the human milk of healthy mothers in Beijing and possessed excellent probiotic properties (Yin et al., 2019). Both strains are known as effective probiotics that are closely associated with human health and are commonly used in functional foods and for fermentation in the dairy industry (Selle and Klaenhammer, 2013; Seddik et al., 2017). Probiotics in human milk may be the direct source of the intestinal microbiota of infants and young children, indirectly affecting their health. *Poria cocos* is a commonly used material in traditional Chinese medicine with various biological activities, such as anti-tumor, immune regulation, anti-inflammation, anti-oxidation, and anti-aging effects. Although probiotics are functional microorganisms that transform animal and plant materials *via* fermentation, improve the bioavailability of nutrients, and enrich the sensory quality of foods, no study has examined the interaction of human-derived probiotics and medicinal materials (Tamang et al., 2016). Therefore, the present study aimed to explore the interaction between human milk-derived probiotics and *Poria cocos*, the prebiotic effects of this traditional Chinese medicine, and the probiotic potential of human milk-derived probiotics.

To explore the proliferation-enhancing effect of *Poria cocos* on probiotics and to determine its optimal concentration, four concentrations of *Poria cocos* fermentation broth (1, 2.5, 5, and 7.5%) were analyzed. Usually, the concentration of fermentation additives is optimal at 1–10%, and excessive addition may affect the organoleptic properties of fermented products. By comparing the OD value of the two probiotics in *Poria cocos* fermentation broth at the tested concentrations 20 h after the onset of fermentation (Table 1), we found that the proliferation-enhancing effect of *Poria cocos* on *L. plantarum* R9 and *L. gasseri* B1-27 was correlated with the concentration of *Poria cocos*. When the concentration of *Poria cocos* in the medium was 2.5%, the fermentation broths of the probiotic strains reached their maximum OD values and increasing the concentration of *Poria cocos* in the fermentation broth led to decreasing OD values of the fermentation broths. When the concentration of *Poria cocos* reached 7.5%, the growth of *L. plantarum* R9 and *L. gasseri* B1-27 was inhibited. This might be because *Poria cocos* itself contains a large number of polysaccharides, small molecular sugars, amino acids, and a variety of trace elements, which might serve as a carbon and nitrogen sources for the probiotic strains, thus promoting the growth of the probiotic bacteria. It is also possible that when the concentration of *Poria cocos* reached 7.5%, the inhibitory factors present in *Poria cocos* exceeded the beneficial effects. Due to the high concentration of saccharides in the solution, the internal and external osmotic pressure imbalance of the bacteria inhibited their proliferation. The above results show that, in a certain concentration range, *Poria cocos* significantly enhances the proliferation of *L. plantarum* R9 and *L. gasseri* B1-27, and a 2.5% *Poria cocos* concentration yielded an optimal effect. However, the concentration range used in this study was still wide, and subsequent, more accurate optimization is warranted for the future production and application of *Poria cocos*.

**Figure 6:**
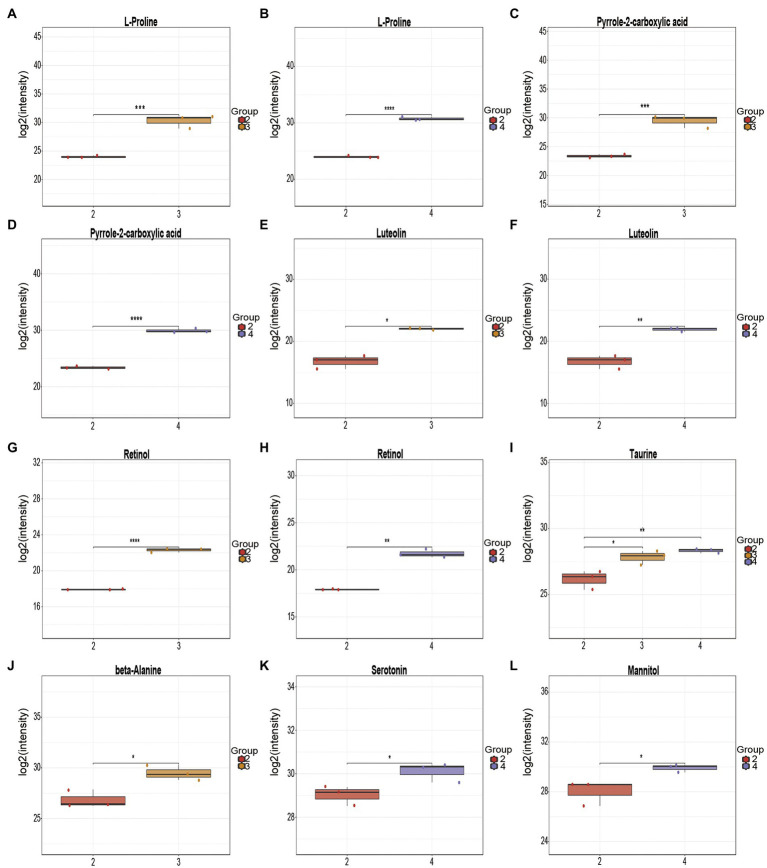
Box plot of differential metabolite changes before and after fermentation. Changes in L-proline after **(A)** *Lactobacillus plantarum* R9 and **(B)** *Lactobacillus gasseri* B1-27 fermentation. **(C)** Changes in pyrrole-2-carboxylic acid after fermentation **(C)** *L. plantarum* R9 and **(D)** *L. gasseri* B1-27 fermentation. Changes in luteolin after **(E)** *L. plantarum* R9 and **(F)** *L. gasseri* B1-27 fermentation. Changes in retinol **(G)** *L. plantarum* R9 and **(H)** *L. gasseri* B1-27 fermentation. **(I)** Changes in taurine after *L. plantarum* R9 and *L. gasseri* B1-27 fermentation. **(J)** Changes in beta-alanine after *L. plantarum* R9 fermentation. **(K)** Changes in serotonin after *L. gasseri* B1-27 fermentation. **(L)** Changes in mannitol after *L. gasseri* B1-27 fermentation.

Based on LC–MS non-targeted metabolomics, 35 and 45 differential metabolites were identified from the fermentation broth of *Poria cocos* before and after fermentation with *L. plantarum* R9 and *L. gasseri* B1-27 (Figure 6). The differential amino acids identified from the *Poria cocos* fermentation broth of *L. plantarum* R9 included L-proline, beta-alanine, taurine, N-acetylleucine, and N-acetyl leucine. These free amino acids and peptides with various physiological functions are important contributors to the unique organoleptic and nutritional quality of *Lactobacillus* sp. fermented foods (Hu et al., 2022). For instance, L-proline has been tested for its anti-tumor activity in tissue culture and *in vivo*. As one of the most popular sport supplements worldwide, beta-alanine can increase carnosine content in skeletal muscle and act as an intracellular pH buffer, improving exercise performance and delaying exercise fatigue (Trexler et al., 2015). Taurine is a sulfur-containing non-essential amino acid that helps regulate cell volume and anti-oxidant defense against stress, and plays an important role in cellular redox homeostasis and skeletal muscle function (Spriet and Whitfield, 2015); it also has physiological effects in regulating lipid metabolism disorders and lowering blood glucose, which has been confirmed in both animal and clinical studies (Kp and Martin, 2022). Probiotics can produce a variety of organic acids during fermentation, thereby reducing the pH of the fermentation broth and effectively inhibiting the production of miscellaneous bacteria during fermentation. For instance, the significantly elevated stearic acid levels may help soften blood vessels and prevent hypertension and cardiovascular problems, thereby promoting health (Valenzuela et al., 2011). With an electron-rich pyrrole ring and a 2-carboxylic acid functional group, pyrrole-2-carboxylic acid can provide a nucleophilic center, endowing it with anti-bacterial, anti-fungal, anti-inflammatory, and anti-tumor activities (Hu et al., 2020). Retinol is a dietary compound with vitamin A activity. Vitamin A is a key nutrient in the human diet, with particular importance in vision maintenance, embryonic development, immunity, tissue repair, and homeostasis (O’Connor et al., 2022). Flavonoids are a large class of secondary metabolites with diverse structures and widely distributed in plants. Some flavonoids have diverse structures and some of them have important pharmacological activities such as anti-cancer and anti-inflammation (Middleton et al., 2000). They can also add a pleasant aroma to fermented products. Among the differential metabolites identified in the present study, luteolin has high medicinal value. Compared with other flavonoids, luteolin is most effective in inhibiting tumor cell proliferation (Seelinger et al., 2008), and it can also protect the nervous system, reduce the occurrence of neuropathy, and improve memory and cognitive function through its anti-oxidant and anti-inflammatory effects (Yao et al., 2021). These differential metabolites, which were significantly increased after fermentation, can potentially enhance the health benefits of the final product (Tables 3, 4).

**Table 3:** Metabolomics sample group information.

Group	Group name	Abbreviation	Quantity
1	Initial state	Chu	3
2	After sterilization (before fermentation)	Mie	3
3	After *L. plantarum* R9 fermentation	R	3
4	After *L. gasseri* B1-27 fermentation	B	3

**Table 4:** Differential metabolites between the four tested groups.

Group	Total	Up-regulated	Down-regulated	Total differential metabolites
2 versus 1	380	5	14	19
3 versus 2	380	30	5	35
4 versus 2	380	36	9	45
4 versus 3	380	5	13	18

Other types of organic acids, amino acids, and alcohols were also produced as a result of the co-fermentation of *L. gasseri* B1-27 and *Poria cocos* powder. In addition to L-proline, taurine, pyrrole-2-carboxylic acid, luteolin, and retinol, serotonin was also produced. This monoamine can improve people’s mood, memory, sleep, and thermoregulation, and has anti-inflammatory and immune-enhancing properties (Balakrishna et al., 2021; Ślifirski et al., 2021). Mannitol, a naturally occurring six-carbon sugar alcohol, is a natural sweetener with low glycemic index. It has a wide range of applications in the food and pharmaceutical industries and can also act as an antioxidant. Many homologous and heterologous flactic acid bacteria and yeasts used in fermentation are known to produce mannitol (Saha and Racine, 2011), and the use of biological cultures for producing mannitol has advantages compared to its production *via* phytochemical synthesis (Chen et al., 2020). In the future, mannitol might be produced by co-fermentation of *L. gasseri* B1-27 and *Poria cocos* powder, and directly applied in food manufacturing, conferring additional nutritional value to fermented products (Martínez-Miranda et al., 2022). The metabolic change process of *Poria cocos* fermentation broth is regulated by a variety of substances and reactions. It is impossible to evaluate all metabolic changes based on a single substance. Thus, metabolic pathway analysis and comparisons against the KEGG database should be performed, followed by metabolite classification according to the pathways or functions the differential metabolites are associated with. The impact of human milk-derived probiotics on the biological metabolism of *Poria cocos* fermentation broth can then evaluated macroscopically. As shown in Figure 4, the number of metabolites associated with pyrimidine metabolism, phosphopentose, yeast meiosis, ABC transporter, and insulin signaling pathways increased most significantly after the co-fermentation of *L. plantarum* R9 and *Poria cocos*. The number of metabolites related to ABC transporters, cancer center carbon metabolism, mineral absorption, pyrimidine metabolism, and yeast meiosis signaling pathway increased most significantly after the co-fermentation of *L. gasseri* B1-27 with *Poria cocos*. The pyrimidine metabolic pathway affects the synthesis of important substances, such as nucleotides and amino acids, in *Poria cocos*. ABC transporters (ATP-binding cassette transporters) are a large and ancient family of transmembrane transport proteins, which are involved in physiological activities such as the transport and accumulation of metabolites, detoxification of harmful substances, stomatal regulation, and plant defense (Zhang et al., 2022). The pentose phosphate pathway is an important pathway of sugar metabolism in plants. Its metabolites include NADPH, which is involved in biosynthesis, and pentose phosphate, which is required for nucleic acid metabolism, while some intermediate products can participate in amino acid and fatty acid synthesis (Patra and Hay, 2014).

Probiotic co-fermentation has been found to promote the production of metabolites with functional properties that lead to beneficial changes in fermented products. Co-fermentation experiments of two lactic acid bacteria promoted the formation of volatile metabolites, especially acetaldehyde, diacetyl, and acetoin, and increased the quantities of flavor compounds in yogurt (Tian et al., 2019). Additionally, the co-fermentation of oat milk by *Lactobacillus* and *Bifidobacterium* decreased the levels of both lignans and phytic acid, while increased the levels of some polyphenols and the bioaccessibility of specific amino acids, vitamins, and polyphenols (Bocchi et al., 2021). Studies on two yeasts showed that co-fermentation adjusted the content of polyphenols and ethyl ester to decrease the bitterness, and enhance the aroma of cider (Wang N. et al., 2022) and wine (Hu et al., 2018). In addition, co-fermented yeast and *Lactiplantibacillus plantarum* enhanced the quality and preservation of fermented teas (Wang R. et al., 2022). No studies have focused on the eventual possibility of the production of metabolites that may be deleterious to human health. In the future, research in the field of co-fermentation is likely to receive increasing attention. However, animal experiments may be needed to further investigate the active ingredients and efficacy of the metabolites produced post fermentation.

Overall, we determined that the optimal concentration of *Poria cocos* to be used in co-fermentation was 2.5% and identified more than 35 differentially expressed metabolites in each probiotic bacteria after co-fermentation. Moreover, several beneficial metabolites were significantly elevated as a result of the co-fermentation process indicating the valuable role of *Poria cocos* as a functional food. Furthermore, the present study lays the foundation for further research on infant formula and other functional prebiotic fermented products, providing a reference for using probiotics and medicinal and edible homologous functional plant metabolites as food additives.

## Data availability statement

The original contributions presented in the study are included in the article/supplementary material, further inquiries can be directed to the corresponding author.

## Author contributions

QW: formal analysis, writing—original draft, visualization, validation, and methodology. LC: conceptualization and funding acquisition. KY and XW: writing—reviewing and editing. WQ: related technical guidance and format. All authors contributed to the article and approved the submitted version.

## Funding

The present study was funded by the National Natural Science Foundation of China (grant no. 32072191) and the Beijing Innovation Team of Livestock Industry Technology System, Daxing District Major Scientific and Technological Achievements Transformation Project (grant no. 2020006).

## Conflict of interest

Authors QW, KY, XW, WQ, and LC were employed by Beijing Sanyuan Foods Co. Ltd.

## Publisher’s note

All claims expressed in this article are solely those of the authors and do not necessarily represent those of their affiliated organizations, or those of the publisher, the editors and the reviewers. Any product that may be evaluated in this article, or claim that may be made by its manufacturer, is not guaranteed or endorsed by the publisher.

## Footnotes

^1^ https://hmdb.ca

^2^ https://massbank.eu/MassBank/

^3^ https://www.lipidmaps.org

^4^ https://www.mzcloud.org

^5^ https://www.genome.jp/kegg/

## References

[ref1] Balakrishna P. George S. Hatoum H. Mukherjee S. (2021). Serotonin pathway in cancer. Int. J. Mol. Sci. 22:1268. doi: 10.3390/ijms22031268, PMID: 33525332 PMC7865972

[ref2] Binda S. Hill C. Johansen E. Obis D. Pot B. Sanders M. E.. (2020). Criteria to qualify microorganisms as “probiotic” in foods and dietary supplements. Front. Microbiol. 11:1662. doi: 10.3389/fmicb.2020.01662, PMID: 32793153 PMC7394020

[ref3] Bocchi S. Rocchetti G. Elli M. Lucini L. Lim C.-Y. Morelli L. (2021). The combined effect of fermentation of lactic acid bacteria and in vitro digestion on metabolomic and oligosaccharide profile of oat beverage. Food Res. Int. 142:110216. doi: 10.1016/j.foodres.2021.110216, PMID: 33773694

[ref4] Boulesteix A. L. Strimmer K. (2007). Partial least squares: a versatile tool for the analysis of high-dimensional genomic data. Brief. Bioinform. 8, 32–44. doi: 10.1093/bib/bbl016, PMID: 16772269

[ref5] Chen M. Zhang W. Wu H. Guang C. Mu W. (2020). Mannitol: physiological functionalities, determination methods, biotechnological production, and applications. Appl. Microbiol. Biotechnol. 104, 6941–6951. doi: 10.1007/s00253-020-10757-y, PMID: 32601737

[ref6] Dunn W. B. Broadhurst D. Begley P. Zelena E. Francis-Mcintyre S. Anderson N.. (2011). Procedures for large-scale metabolic profiling of serum and plasma using gas chromatography and liquid chromatography coupled to mass spectrometry. Nat. Protoc. 6, 1060–1083. doi: 10.1038/nprot.2011.335, PMID: 21720319

[ref7] Gueimonde M. Delgado S. Mayo B. Ruas-Madiedo P. Margolles A. de los Reyes-Gavilán C. G. (2004). Viability and diversity of probiotic lactobacillus and Bifidobacterium populations included in commercial fermented milks. Food Res. Int. 37, 839–850. doi: 10.1016/j.foodres.2004.04.006

[ref8] Heikkilä M. P. Saris P. E. J. (2003). Inhibition of Staphylococcus aureus by the commensal bacteria of human milk. J. Appl. Microbiol. 95, 471–478. doi: 10.1046/j.1365-2672.2003.02002.x, PMID: 12911694

[ref9] Hu K. Jin G. J. Mei W. C. Li T. Tao Y. S. (2018). Increase of medium-chain fatty acid ethyl ester content in mixed *H. uvarum*/*S. cerevisiae* fermentation leads to wine fruity aroma enhancement. Food Chem. 239, 495–501. doi: 10.1016/j.foodchem.2017.06.151, PMID: 28873596

[ref10] Hu Q. Wang S. Zhou Y. Fu H. (2020). Advances in biosynthesis and biological activity of pyrrole-2-carboxylic acid. Biochem. Eng. 6, 157–160. doi: 10.3969/j.issn.2096-0387.2020.06.043

[ref11] Hu Y. Zhang L. Wen R. Chen Q. Kong B. (2022). Role of lactic acid bacteria in flavor development in traditional Chinese fermented foods: a review. Crit. Rev. Food Sci. Nutr. 62, 2741–2755. doi: 10.1080/10408398.2020.1858269, PMID: 33377402

[ref12] Kp A. D. Martin A. (2022). Recent insights into the molecular regulators and mechanisms of taurine to modulate lipid metabolism: a review. Crit. Rev. Food Sci. Nutr. 1–13, 1–13. doi: 10.1080/10408398.2022.2026873, PMID: 35040723

[ref13] Lu Q. Lu H. Liu X. (2014). Research advance on the multiplication things of probiotics. Food Res Dev 35, 132–136. doi: 10.3969/j.issn.1005-6521.2014.16.033

[ref14] Martín V. Maldonado-Barragán A. Moles L. Rodriguez-Baños M. Del Campo R. D. Fernández L.. (2012). Sharing of bacterial strains between breast milk and infant feces. J. Hum. Lact. 28, 36–44. doi: 10.1177/0890334411424729, PMID: 22267318

[ref15] Martínez-Miranda J. G. Chairez I. Durán-Páramo E. (2022). Mannitol production by heterofermentative lactic acid bacteria: a review. Appl. Biochem. Biotechnol. 194, 2762–2795. doi: 10.1007/s12010-022-03836-5, PMID: 35195836

[ref16] Middleton E. Kandaswami C. Theoharides T. C. (2000). The effects of plant flavonoids on mammalian cells: implications for inflammation, heart disease, and cancer. Pharmacol. Rev. 52, 673–751. PMID: 11121513

[ref17] Mozzi F. Ortiz M. E. Bleckwedel J. De Vuyst L. Pescuma M. (2013). Metabolomics as a tool for the comprehensive understanding of fermented and functional foods with lactic acid bacteria. Food Res. Int. 54, 1152–1161. doi: 10.1016/j.foodres.2012.11.010

[ref18] Navarro-Reig M. Jaumot J. García-Reiriz A. Tauler R. (2015). Evaluation of changes induced in rice metabolome by cd and cu exposure using LC-MS with XCMS and MCR-ALS data analysis strategies. Anal. Bioanal. Chem. 407, 8835–8847. doi: 10.1007/s00216-015-9042-2, PMID: 26403240

[ref19] Niu S. Hao L. Zhao S. Chen Q. (2012). Research progress in polysaccharides from *Poria cocos*. Food Sci. 33, 348–353. doi: 10.7506/spkx1002-6630-201213073

[ref20] O’Connor C. Varshosaz P. Moise A. R. (2022). Mechanisms of feedback regulation of vitamin a metabolism. Nutrients 14:1312. doi: 10.3390/nu14061312, PMID: 35334970 PMC8950952

[ref21] Patra K. C. Hay N. (2014). The pentose phosphate pathway and cancer. Trends Biochem. Sci. 39, 347–354. doi: 10.1016/j.tibs.2014.06.005, PMID: 25037503 PMC4329227

[ref22] Pothuraju R. Sharma R. K. Chagalamarri J. Kavadi P. K. Jangra S. (2015). Influence of milk fermented with lactobacillus rhamnosus NCDC 17 alone and in combination with herbal ingredients on diet induced adiposity and related gene expression in C57BL/6J mice. Food Funct. 6, 3576–3584. doi: 10.1039/c5fo00781j, PMID: 26327356

[ref23] Prado F. C. Parada J. L. Pandey A. Soccol C. R. (2008). Trends in non-dairy probiotic beverages. Food Res. Int. 41, 111–123. doi: 10.1016/j.foodres.2007.10.010

[ref24] Prosekov A. Y. Dyshlyuk L. S. Milenteva I. S. Pavsky V. A. Ivanova S. A. Garmashov S. Y. (2018). Study of the BIOFUNCTIONAL properties of cedar pine oil with the use of testing cultures. Foods Raw Mater. 6, 136–143. doi: 10.21603/2308-4057-2018-1-136-143

[ref25] Saha B. C. Racine F. M. (2011). Biotechnological production of mannitol and its applications. Appl. Microbiol. Biotechnol. 89, 879–891. doi: 10.1007/s00253-010-2979-3 21063702

[ref26] Seddik H. A. Bendali F. Gancel F. Fliss I. Spano G. Drider D. (2017). *Lactobacillus plantarum* and its probiotic and food potentialities. Probiotics Antimicrob. Proteins. 9, 111–122. doi: 10.1007/s12602-017-9264-z 28271469

[ref27] Seelinger G. Merfort I. Wölfle U. Schempp C. M. (2008). Anti-carcinogenic effects of the flavonoid luteolin. Molecules 13, 2628–2651. doi: 10.3390/molecules13102628, PMID: 18946424 PMC6245397

[ref28] Selle K. Klaenhammer T. R. (2013). Genomic and phenotypic evidence for probiotic influences of *Lactobacillus gasseri* on human health. FEMS Microbiol. Rev. 37, 915–935. doi: 10.1111/1574-6976.12021, PMID: 23488471

[ref29] Ślifirski G. Król M. Turło J. (2021). 5-HT receptors and the development of new antidepressants. Int. J. Mol. Sci. 22:9015. doi: 10.3390/ijms22169015, PMID: 34445721 PMC8396477

[ref30] Smith C. A. Want E. J. O’Maille G. Abagyan R. Siuzdak G. (2006). XCMS: processing mass spectrometry data for metabolite profiling using nonlinear peak alignment, matching, and identification. Anal. Chem. 78, 779–787. doi: 10.1021/ac051437y, PMID: 16448051

[ref31] Spriet L. L. Whitfield J. (2015). Taurine and skeletal muscle function. Curr. Opin. Clin. Nutr. Metab. Care 18, 96–101. doi: 10.1097/MCO.0000000000000135 25415270

[ref32] Sukhikh S. A. Astakhova L. A. Golubcova Y. V. Lukin A. A. Prosekova E. A. Milenteva I. S.. (2019). Functional dairy products enriched with plant ingredients. Foods Raw Mater. 7, 428–438. doi: 10.21603/2308-4057-2019-2-428-438

[ref33] Tamang J. P. Shin D. H. Jung S. J. Chae S. W. (2016). Functional properties of microorganisms in fermented foods. Front. Microbiol. 7:578. doi: 10.3389/fmicb.2016.00578, PMID: 27199913 PMC4844621

[ref34] Thévenot E. A. Roux A. Xu Y. Ezan E. Junot C. (2015). Analysis of the human adult urinary metabolome variations with age, body mass index, and gender by implementing a comprehensive workflow for univariate and OPLS statistical analyses. J. Proteome Res. 14, 3322–3335. doi: 10.1021/acs.jproteome.5b00354, PMID: 26088811

[ref35] Tian H. Shi Y. Zhang Y. Yu H. Mu H. Chen C. (2019). Screening of aroma-producing lactic acid bacteria and their application in improving the aromatic profile of yogurt. J. Food Biochem. 43:e12837. doi: 10.1111/jfbc.12837, PMID: 31608476

[ref36] Trexler E. T. Smith-Ryan A. E. Stout J. R. Hoffman J. R. Wilborn C. D. Sale C.. (2015). International society of sports nutrition position stand: Beta-alanine. J. Int. Soc. Sports Nutr. 12:30. doi: 10.1186/s12970-015-0090-y, PMID: 26175657 PMC4501114

[ref37] Trygg J. Wold S. (2002). Orthogonal projections to latent structures (O-PLS). J. Chemometrics. 16, 119–128. doi: 10.1002/cem.695

[ref38] Valenzuela A. Delplanque B. Tavella M. (2011). Stearic acid: a possible substitute for trans fatty acids from industrial origin. Grasas Aceites 62, 131–138. doi: 10.3989/gya.033910

[ref39] Wang R. Sun J. Lassabliere B. Yu B. Liu S. Q. (2022). UPLC-Q-TOF-MS based metabolomics and chemometric analyses for green tea fermented with saccharomyces boulardii CNCM I-745 and Lactiplantibacillus plantarum 299V. Curr. Res. Food Sci. 5, 471–478. doi: 10.1016/j.crfs.2022.02.012, PMID: 35252880 PMC8892000

[ref40] Wang N. Zhu Y. Zhu R. Xiao Y. Qiu J. Wu Y.. (2022). Revealing the co-fermentation of Saccharomyces cerevisiae and schizosaccharomyces pombe on the quality of cider based on the metabolomic and transcriptomic analysis. LWT 168:113943. doi: 10.1016/j.lwt.2022.113943

[ref41] Want E. J. Masson P. Michopoulos F. Wilson I. D. Theodoridis G. Plumb R. S.. (2013). Global metabolic profiling of animal and human tissues via UPLC-MS. Nat. Protoc. 8, 17–32. doi: 10.1038/nprot.2012.135, PMID: 23222455

[ref42] Wu F. Li S. J. Dong C. H. Dai Y. C. Papp V. (2020). The genus Pachyma (Syn. Wolfiporia) reinstated and species clarification of the cultivated medicinal mushroom “Fuling” in China. Front. Microbiol. 11:590788. doi: 10.3389/fmicb.2020.590788(J.2022Wolfiporia) 33424793 PMC7793888

[ref43] Xia J. Wishart D. S. (2011). Web-based inference of biological patterns, functions and pathways from metabolomic data using Metabo analyst. Nat. Protoc. 6, 743–760. doi: 10.1038/nprot.2011.319, PMID: 21637195

[ref44] Xu J. Hu F. L. Wang W. Wan X. C. Bao G. H. (2015). Investigation on biochemical compositional changes during the microbial fermentation process of Fu brick tea by LC-MS based metabolomics. Food Chem. 186, 176–184. doi: 10.1016/j.foodchem.2014.12.045, PMID: 25976808

[ref45] Xu D. Tan C. Zheng H. Zhang F. Hou F. Dai X.. (2022). Progress in research on the bioactive component pachymic acid from *Poria cocos*. Food Sci. 43, 273–280. doi: 10.7506/spkx1002-6630-20210826-347

[ref46] Yao X. Ning H. Huang H. Huang L. Wang S. Li Y.. (2021). Research progress on pharmaceutical preparation of luteolin. Chin. Trad. Herb. Drugs. 52, 873–882. doi: 10.7501/j.issn.0253-2670.2021.03.032

[ref47] Yin C. Li Z. Jiang T. Liu L. Liu Y. Wang D.. (2019). Screening and identification of ability lactic acid bacteria from human breast milk and preliminary determination of its ability to hypotensive. Food Sci. Technol. 44, 18–22. doi: 10.13684/j.cnki.spkj.2019.08.004

[ref48] Zelena E. Dunn W. B. Broadhurst D. Francis-McIntyre S. Carroll K. M. Begley P.. (2009). Development of a robust and repeatable UPLC-MS method for the long-term metabolomic study of human serum. Anal. Chem. 81, 1357–1364. doi: 10.1021/ac8019366, PMID: 19170513

[ref49] Zha M. Li K. Zhang W. Sun Z. Kwok L.-Y. Menghe B.. (2021). Untargeted mass spectrometry-based metabolomics approach unveils molecular changes in milk fermented by *Lactobacillus plantarum* P 9. LWT Food Sci. Technol. 140:110759. doi: 10.1016/j.lwt.2020.110759

[ref50] Zhang T. Zhang X. Wang D. Xu X. (2022). The effect of polysaccharide of *Poria cocos* on key metabolites of Bifidobacterium BB-12. Sci. Technol. Food Ind. 43, 24–33. doi: 10.13386/j.issn1002-.(2022).0306.2021110200

[ref51] Zhao Z. Xu C. An M. Wei X. Liao N. Ni Y.. (2019). Isolation, screening and probiotic characteristics analysis of Bifidobacterium from breast milk of Uygur women in Kashi, Xinjiang. Food Sci. 40, 185–192. doi: 10.7506/spkx1002-6630-20181017-170

